# Risk and reproductive decisions: British Pakistani couples’ responses to genetic counselling

**DOI:** 10.1016/j.socscimed.2011.04.011

**Published:** 2011-07

**Authors:** Alison Shaw

**Affiliations:** Department of Public Health, University of Oxford, Oxford OX2 6HE, United Kingdom

**Keywords:** United Kingdom, British Pakistanis, Genetic risk, Reproductive decisions, Prenatal diagnosis, Religion, Muslims, Ethnicity

## Abstract

How far does ethnicity/culture/religion mediate couples’ responses to genetic risk? This paper examines the responses of 51 British Pakistani couples referred to a genetics clinic in southern England to counselling about recurrence risks for genetic problems in children. It is based on fieldwork conducted between 2000 and 2004 that combined participant observation of genetics consultations with interviews in respondents’ homes. Interviews were conducted with 62 adults in connection with these 51 cases, of which 32 were followed through two or more clinical consultations and 12 through more than one pregnancy. Risk responses were categorized as: taking the risk; postponing; exploring risk management or dismissing the risk as irrelevant to current circumstances. Responses were cross-referenced for associations with the severity of the condition, number of affected and unaffected children, availability of a prenatal test, age, gender, and migration history. I found that most couples were initially risk-takers who already had an unaffected child or children. Couples caring for living children with severe conditions were more likely to postpone. However, the risk responses of 15 couples changed over time, most towards and some away from risk management, reflecting changes in couples’ appreciation of the severity of the condition and their subsequent reproductive experiences. The study highlights the diversity and dynamism of responses within one ethnic group and challenges stereotypes about cultural and religious responses to genetic risk.

## Highlights

► Analyses British Pakistani Muslim couples’ responses to counselling about recurrence risk for genetic problems in children. ► Highlights the importance of couples’ previous reproductive history and their appreciation of the severity of the condition. ► Challenges stereotypes about cultural and religious responses to genetic risk.

## Introduction

For couples who have experienced a genetic problem in a pregnancy or child, or have a family history of a genetic condition, genetic counselling provides information about the likely recurrence of that condition. The risks given vary with the inheritance of the condition and the clinical certainty of diagnosis. For parents of an affected child these are typically 1 in 4 for autosomal recessive conditions and 1 in 2 for inheritable autosomal dominant conditions. Parents are usually advised about risk management in subsequent pregnancies. Prenatal genetic tests, carrying a 1% risk of spontaneous abortion, with the option of ending an affected pregnancy, are available for some conditions.

Risk can be defined as uncertainty about a future event that may, on the basis of empirical evidence, be expressed as a probability or frequency (Gigerenzer, 2008). In the context of reproductive decisions, a numerical risk is a statement of uncertainty that, for couples, reduces to a binary: the child will have the condition, or it will not. The question then is whether couples feel the risk is worth taking. Social science research on risk perception shows that much more is at stake than scientifically-derived numerical information because social circumstances and moral considerations determine risk acceptability (Caplan, 2000; Douglas, 1992; Douglas & Wildavsky, 1982; Jenkins, Jessen, & Steffaen, 2005; Lupton, 1999). Thus, reproductive risk is likely to be negotiated not in isolation but in relation to other risks, including those of which actors may not be consciously aware. Risk acceptability is likely to be mediated by such factors as the availability of a prenatal genetic test for the condition, the nature and severity of the condition and the therapeutic possibilities available for it, and attitudes to abortion.

Attitudes to the use of prenatal diagnosis and abortion in western populations with elevated risk for conditions such as cystic fibrosis, breast cancer and fragile X are significantly influenced by the severity of the condition for which there is risk, the therapeutic possibilities for it and the child’s quality of life (Lafayette, Abuelo, Passero, & Tantrvahi, 1999; Lodder et al., 2000; Skinner, Sparkman, & Bailey Jr., 2003). In western and non-western settings, parents are more likely to consider abortion for conditions resulting in infant or childhood death or associated with severe intellectual and physical disability (Arif et al., 2008; Mansfield, Hopfer, & Marteau, 1999; Shaffer, Caughey, & Norton, 2006; Zlotogora, 2002).

Religion has been identified as a potentially critical influence on attitudes to prenatal testing and abortion for haemoglobinopathies in the Middle East and in Pakistan. For some of the Lebanese Muslims in one study prenatal testing raised concerns about the religious acceptability of abortion for foetal abnormalilty (Zahed & Bou-Dames, 1997). In studies in Pakistan and Saudi Arabia, couples who initially opposed or were hesitant about prenatal testing and abortion on religious grounds were relieved when informed about Islamic opinions permitting abortion within the first 120 days of conception in cases of foetal abnormality (Ahmed et al., 2000; Alkuraya & Kilani, 2001). Across the Middle East, religious rulings on the permissibility of prenatal diagnosis, selective abortion and other new reproductive technologies such as IVF, gamete donation and surrogacy have taken diverse forms, sometimes constraining choice and sometimes offering new opportunities; there is considerable heterogeneity in the ways in which couples negotiate the possibilities even within a single religious tradition (Clarke, 2009; Inhorn, 2003, 2006; Khan, 2000).

Having an affected child or children may significantly influence responses to risk information. Parents already caring for a living child with a genetic condition may be more likely to postpone or avoid another pregnancy, feeling unable to manage another child, especially if they already have other children (D’Amico, Jacoponi, Vivona, & Frontali, 1992). Those already caring for affected children may be deeply ambivalence towards prenatal testing and abortion, choosing to avoid confronting the associated moral dilemmas by avoiding future pregnancies or, if they go ahead, by declining prenatal testing or by limiting testing to ‘for information only’ (Kelly, 2009). Alternatively they may choose abortion to avoid another affected child’s suffering (Ahmed, Green, & Hewison, 2006). The death of an affected child may enable couples to attempt to try again, the death perhaps removing the burden of caring and ending parents’ uncertainties about their ability to manage another child (Lippman-Hand & Fraser, 1979). Reproductive choices are thus not necessarily static but can alter with experience. Parents’ decisions may change over time because of developments in the therapeutic possibilities for the disorder in question and because a DNA test has become available.

Another strand of research examines parental responses to genetic risk in the context of debates about how far women are being drawn into the medicalization/geneticization of reproduction and health in the modern world (see e.g. Lock & Kaufert, 1998; Press & Browner, 1997; Taussig, 2009). Women who refuse prenatal testing may employ the concepts and logic of modern forms of risk assessment when declining tests, and hold views on the use of prenatal tests and abortion not significantly different from those of women who accept these technologies: the reasoning associated with accepting or refusing prenatal screening reflects different points along a continuum, rather than morally distinct categories (Markens, Browner, & Press, 1999). Parents of living children with genetic conditions for which prenatal genetic tests are available who decide to postpone another pregnancy, or proceed but decline a prenatal genetic test, or who limit testing to ‘for information only’, are responding with a strategy of responsible parenting to the medical uncertainty associated with using reproductive technologies by ‘choosing not to choose’ between different prenatal testing options (Kelly, 2009).

This paper adds to these areas of research by examining British Pakistani-origin couples' responses to counselling about the recurrence risk of genetic problems in children. The study asks two main questions. First, do couples feel the risk is worth taking, and if not, how do they negotiate it? Secondly, what is at stake in couples’ responses to risk: what is the impact of such factors as parents’ views of the severity of the condition and the child’s quality of life; their religious beliefs, age, generation, education, the influence of gender norms, patriarchal attitudes, migration history, and the experience of having affected or unaffected children? The study also asks if British Pakistani parents’ responses to risk change over time, and if so why. It concludes by addressing the broader question of whether couples of British Pakistani ethnicity are any more or less engaged than any other group in negotiating with modern medical genetic risk assessment and management. Since the focus here is on one ethnic group, an initial overview of research concerning ethnicity and responses to genetic risk is provided.

### Ethnicity and risk

In western populations, ethnicity usually refers to minorities of relatively recent non-white immigrant origin, such as Hispanics in the USA or South Asians in the UK. The term has context-dependent meanings that often imply essentialised cultural or religious difference. In understanding health differentials, attempts have been made to dissociate the various factors commonly linked with ethnicity, such as socio-economic status, education level, language use, religion and cultural attitudes concerning gender and authority. Research has examined, for example, the effects of faith on medical helpseeking (Greil et al., 2010; Laird, Amer, Barnett, & Barnes, 2007; Mir & Sheikh, 2010; Rozario, 2005). Religion has been implicated in apparent ethnic or racial differences in uptake of genetic prenatal screening and testing for Down syndrome and haemoglobinopathies (Kuppermann, Gates, & Washington, 1996; Rapp, 1998; Rowe, Garcia, & Davidson, 2004). Recent studies, however, challenge the assumption that ethnicity reflects deep-seated and non-negotiable cultural attitudes and/or religious beliefs, for example that Muslims will decline prenatal testing and abortion because of their religious convictions. Rather, there are substantial variations within ethnic and faith groups in prenatal testing decisions and more similarities than differences between them (Browner, Preloran, & Cox, 1999; Learman et al., 2003; Saucier et al., 2005). Across ethnic groups, the consequences of the condition for the child’s quality of life is a critical consideration; religious belief is negotiable and contingent; and reproductive decisions are considered to be personal, and reflect social and family circumstances (Ahmed, Atkin, Hewison, & Green, 2006; Ahmed, Green et al., 2006; Ahmed et al., 2008; Atkin, Ahmed, Hewison, & Green, 2008; Hewison et al., 2007).

Ethnic differences in the uptake of screening and testing for genetic conditions can be age-related, the attitudes of women over 35 years of age being more likely to reflect faith, fatalism and concerns about miscarriage risks (Kuppermann et al., 2006; Shaffer et al., 2006). Ethnic differences in service uptake without significant attitudinal differences may reflect constraints on ethnic minority women making decisions in line with attitudes, rather than deep-rooted cultural or religious differences (Dormandy, Michie, Hooper, & Marteau, 2005); constraints may include not receiving information from health professionals who assume there is no point offering prenatal diagnosis to Muslim women because Islam forbids termination of pregnancy and professional disempowerment in the face of ethnic diversity (Kai et al., 2007; Modell et al., 2000). Service uptake may also be constrained by age, language use and level of gender emancipation (Fransen et al., 2010). A comparison of Palestinians in the USA and in Palestine identifies cultural differences in patients’ expectations of directive rather than non-directive counselling and in the degree of women’s autonomy in prenatal decision-making (Awwad, Veach, Bartels, & LeRoy, 2008).

## Methods

### Sample and recruitment

Research participants were recruited from the Pakistani referrals to an NHS genetics clinic in High Wycombe, in southern England, their ethnicity inferred from their names. High Wycombe’s Pakistani population of 9303 (2001 Census) comprises older pioneer-generation immigrants of the post-war era and their descendants. Approximately two genetics clinic referrals per month concern Pakistani-origin families. From 76 Pakistani-origin clinic referrals a total of 66 cases were recruited the project over three years after receiving an invitation letter, in Urdu and English, from the consultant geneticist, further information about the project from the researcher and giving their written consent. The research was conducted between 2000 and 2004 and supported by a project grant from the Wellcome Trust UK (GR063078MA). Ethics approval was obtained from the South Buckinghamshire NHS research ethics committee.

**Fig. 1:**
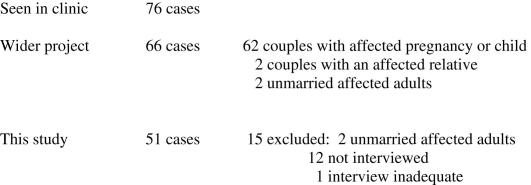
Study recruitment and selection.

Since this study concerns parental responses to recurrence risk, 51 cases were selected from the total of 66 because these concerned married adults who had received recurrence risk information either after having a pregnancy or child with a genetic or probably genetic problem or because of a history of a genetic condition in the family. Fifteen cases were excluded, two because they concerned unmarried, childless adults, and thirteen because interview data were lacking or inadequate: nine interviews were not offered because the cases were seen in the clinic late into the study (7 cases, one of which concerned an unmarried adult) or had involved a recent, distressing termination of pregnancy (2 cases); two interviews were not conducted because the participants moved away; one interview was declined and one was inadequate. Fig. 1 summarises the steps taken in case recruitment and selection.

### Interviews and participant observation

Fieldwork combined participant observation of genetics consultations with interviews, in respondents’ homes, in Urdu or English; the researcher knows both languages.62 adults, 42 women and 20 men, representing the 51 cases were interviewed. In 13 cases, husbands and wives were interviewed together and in ten of these cases they were also interviewed separately. In 28 cases, wives rather than husbands were the main interviewees because husbands were at work and because some couples felt the research topic was ‘women’s business’, a perception probably influenced by the female gender of the researcher. One husband thought it would be therapeutic for his wife to talk to the researcher alone, since she had declined formal bereavement counselling. Nonetheless, the interviewer met and had briefer conversations with all except four husbands at the genetics clinic or in their homes. In 7 cases, husbands rather than wives were interviewed: one wife was deaf, three wives had learning difficulties and three wives were present but deferred to their husbands throughout their interviews. In 3 cases, interviews were conducted with the couple’s relatives: a woman who accompanied her sister to a genetic clinic appointment; the aunt of an affected child whose mother had died and the brother of a woman who was at work all day and whose husband was in Pakistan, awaiting his UK entry permit.

Interviews were semi-structured, following a broad topic guide that included the interviewee’s understandings of the causality of the problem and its inheritance; their views of genetic diagnosis and reproductive risk; their concerns about the affected child (if living); their reproductive ambitions; their views about the medical management of pregnancy and genetic risk, and the impact of the experience of having an affected, child, pregnancy or close relative. The focus of discussion tended to reflect interviewees’ concerns at different stages of the diagnostic process and their reproductive careers. Of the 51 cases, 32 involved more than one clinical consultation over the study period, and more than one interview and clinical observation. In twelve of these 32 cases, couples returned to the genetics clinic for risk advice because of a new pregnancy, so these cases were followed through more than one pregnancy over several years. Interviews were in 31 cases supplemented by conversations with other relatives involved in the care of an affected child, such as the non-interviewed spouse, the interviewee’s parents (the child’s grandparents) or in-laws or siblings. On four occasions, the interviewee’s friends or relatives arrived while interviews were in progress and two interviews overlapped with visits from statutory service staff caring for an affected child.

In twelve cases, following the diagnostic process via participant observation involved the researcher in numerous meetings, telephone conversations and direct clinical negotiations in which she acted as interpreter and mediator. The fact that the researcher knows Urdu as well as English, and is of White British ethnicity, placed her in an unusual position in relation to both the medical staff and the Pakistani patients: the former entrusting her with the translation of clinical information, the latter trusting her to interpret and mediate on their behalf. In recognition of these roles the researcher received honorary clinical contracts from the relevant hospitals. Observational data and all but two interviews were recorded as notes and written-up fully in English later in a field diary and case notes; two interviews were audio-taped and transcribed later, the interviews in Urdu translated into English by the researcher.

## Analysis

Socio-demographic data relating to each case were entered into an excel file to permit simple quantification of variables: parents’ age, gender, education, migration history (as indicated by country and level of education), family origin in Pakistan, consanguinity, number of unaffected and affected pregnancies or children, desire for more children, other affected relatives, and the severity of the condition. The likely mode of inheritance of the condition and the recurrence risk information provided by doctors was also recorded for each case.

Analysis of the qualitative data required familiarity with the data, which was achieved by the researcher reading and re-reading the observational notes and interview transcripts in their entirety to identify key themes relating to risk perception and attitudes to prenatal diagnosis. From this, the researcher developed an overarching thematic framework through which risk responses could be categorized as ‘taking the risk’, implying risk acceptability, or as entailing one of three forms of risk-negotiation, implying non-acceptability. Risk takers were those who proceeded with another pregnancy without seeking additional risk management. Postponers initially delayed another pregnancy. Risk-managers pursued additional techniques for managing risk including prenatal genetic tests, or expressed the desire to utilize these techniques when they did become pregnant. The exempt asserted that risk information was irrelevant to them in their current circumstances. Some couples could be allocated to more than one category of response as their circumstances and experiences changed over the course of the fieldwork. The responses of these couples were therefore analysed further and subcategorized. Responses were cross-referenced for associations with the socio-demographic variables listed above, including reproductive history, severity of the condition and number of affected and unaffected children, and to identify similarities and differences between responses. In the presentation of findings, cases are allocated a Roman alphabet letter that identifies the adults in connection with each case but has no relationship to their real names.

## Findings

After presenting the socio-demographic and medical characteristics of the sample, this section describes the couples’ risk responses and how, for fifteen couples, these had changed by the end of the fieldwork. The case presentation seeks to highlight why the initial responses of more than half of the couples entailed ‘taking the risk’, and why the number of risk managers had more than doubled by the end of the fieldwork. The findings highlight the significance, for parents, of having or not having an unaffected child, their experience of the condition for which there is risk, whether or not they are caring for an affected child, their understanding the severity of the condition and the availability of a prenatal genetic test. All these factors may change over time and with subsequent experience, rendering parents’ risk responses contingent rather than fixed. The discussion section comments further on the relationship between risk responses and the socio-demographic characteristics of the sample.

### Socio-demographic characteristics and migration history

The average maternal age was 31 years and the average paternal age was 34 years. Most parents (74.5% of the women and 70% of the men) were young: aged between 20 and 34 years at the date of the first interview. A minority (25.5% of the women, 30% of the men) of parents were older, aged between 35 and 64 years. All couples had at least one affected child or pregnancy apart from three couples seeking risk information because of a family history. Table 1 presents data on age, gender, education, family origins, parental consanguinity and number of affected and unaffected children.

The older women were born and educated in Pakistan and had all come to Britain to join husbands of the pioneer or second generation. The older men had come to Britain as pioneer-generation labour migrants with minimal or secondary education from Pakistan, or as pre-school, older or teenage sons of pioneer-generation parents.

The younger adults were either UK-raised or Pakistan-raised. Two thirds (64%) of the younger men comprised UK-educated sons or grandsons of pioneer-generation migrants. These men were UK-born or (in 6 cases) had entered the UK as pre-school or older children or teenagers. One third (36%) of the younger men was educated in Pakistan to secondary level or beyond and had, with one exception, come to Britain as marriage migrants. Marriage migration is a common contemporary phenomenon among young British Pakistani adults and usually involves consanguineous relatives (Shaw, 2009).

The majority (74%) of the younger women were Pakistan-educated, most (20/28) to secondary level or beyond and all except two were marriage migrants. The exceptions were a woman who came to Britain as her parents’ dependant, who was joined later by a husband from Pakistan, and a young woman who accompanied her husband to Britain for work. A smaller proportion (26%) of the younger women were UK-born and educated to secondary or higher levels, with husbands from Pakistan. Just one young couple had recently come to the UK solely for the husband’s work and did not fit the pattern of marriage migration.

### Medical characteristics

Table 2 summarises genetic diagnostic and risk information for the 54 conditions identified in the study sample (three families being affected by two conditions). Clinicians informed couples about the most likely risk, even where diagnosis was uncertain. Couples were informed about prenatal risk management via additional ultrasound or prenatal genetic tests if available for the condition.

### Initial risk responses

Table 3a summarises couples’ initial risk responses.

#### Risk-takers

Initially, half the couples were risk-takers. In thirteen cases a British-raised partner expressed scepticism of their risk by associating it with the public health discourse of genetic risk in consanguineous marriages. After his first genetics consultation, one young UK-raised father married to his first cousin said,

I think there is an automatic assumption that it is because you are cousins. Is this the first question they would have asked a white family? … It is the media that has made it into an issue … [but] I don’t think this [issue] changes anything, I would still go ahead and do it (Mr. B, one low-severity affected daughter).

Another young UK-raised father focused on the rarity of the condition, repeating reassuring comments made by the doctors involved in the case:

The neurologist told us it was a one off, unlikely to happen again, “[This] syndrome is very rare, one in 400,000 births”, he said. It was a very worrying pregnancy. There were no [prenatal genetic] tests available. A week before the birth, I asked the gynaecologist, “What is the chance of it happening again?” He said, “It is like me telling you I am going to the moon and back” (Mr. N, one unaffected daughter, one infant death).

The next baby had the same fatal condition but now, quoting his paediatrician, the father hoped his bad luck would be over:

He told us, “You are very unlucky…There is a risk element that it will happen again, but it is okay to try for another baby. The risk is not greater than before. It is unlikely the dice will land again on a six and six”. We really did feel this time that our bad luck is over and it is going to be okay (Mr. N.)

Seven couples had one unaffected child and twelve had more than one before their affected child was born or before receiving risk information. Four parents contextualized their response with reference to their ability to have unaffected children. For example, a young UK-raised mother who had lost two infants to two different recessive conditions recalled being given a recurrence risk that made no sense to her because she already had an unaffected son and daughter:

We were told it goes up to fifty-fifty…What do you do? There’s no chance of a healthy baby – a fifty-fifty one way or the other. Doesn’t that sound scary? We have had the other two children checked and there’s no sign of it. I am still married to the same person. How can they say now that I have a fifty-fifty chance of one or the other, and I have two healthy children? ….We want a large family; my husband says I should just put it behind me and try again (Mrs. L).

Referring to the fact that she had two unaffected daughters before having one and then another son with a low-severity condition, a young Pakistan-raised mother believed ‘this problem only affects girls’, and went on to have another unaffected daughter.

Three couples expressed their desire for another child in terms of wanting to give their child a sibling. For example, after his second baby died from a fatal rare recessive condition for which there is no prenatal test one young UK-raised father recalled:

Perhaps it was selfish, but we wanted him to have a brother or sister to play with. When I get back from work, all he wants to do is play with me, he won’t leave me alone. But I am tired after work. All I want to do is relax, and watch the evening news, but he pesters me for attention. We thought it would be good for him if we had one more child (Mr. J, one unaffected son).

For three couples with an unaffected child or children, the early death of the affected infant seemed to facilitate the decision to try again as if, as one father put it, they ‘were trying to fill that vacuum up, to replace each lost baby’. For one young childless couple, taking the risk and trying again after two miscarriages and an infant death was necessary for sustaining their marriage because the UK-born husband’s parents, in whose house the couple were living, were suggesting their son divorces his Pakistan-raised first cousin; as his wife put it: ‘I need a child. If I had just one living child, it would not be like this’ (Mrs. I, childless).

A prenatal test was unavailable for all but three risk takers. Additional scans and foetal monitoring may provide information about the baby; phenotypic features of some conditions are recognizable from ultrasound scanning, sometimes enabling a diagnosis from clinical signs rather than a genetic test (Shaw, 2003), but not always early enough, or definitively enough, for parents to feel able to end the pregnancy. Doctors’ theoretical discussion of prenatal genetic testing, with the option of ending the pregnancy, raised concerns for these parents about the risk of spontaneous miscarriage and the ethics of abortion. For example, this was an issue for the young Pakistan-raised mother of an affected child with a high-severity condition. This couple took the risk and had another baby, the UK-raised father having interpreted the clinical advice to wait for a prenatal test as being instructed not to have more children because they are cousins. His wife, however, when interviewed in Urdu alone, said she disagreed with her husband and thought that they should use birth control because she did not want another affected child and if a scan showed an affected baby ending the pregnancy would be wrong:

Q: If the child is very handicapped, if, under certain conditions, early in pregnancy. Would it be difficult to decide what to do?

A: For you, for your people, you might have a difficult decision to make. But for us, the problem is solved before we even go to the clinic. The solution is already there. Our Koran says that in every circumstance to stop a pregnancy is wrong, unless the pregnancy stops by itself, naturally. (Mrs K, one unaffected son.)

Three young couples were offered a prenatal test and declined it. Two had living affected relatives, knew that some treatments are available for managing the condition, and considered abortion unacceptable. The couple who declined a prenatal genetic test had no prior experience of the condition and believed the doctors might be wrong about the abnormalities observed by ultrasound and that their child might be unaffected; ‘These things are in the hands of God’, the father said (Mr. P, one unaffected son, one severely-affected pregnancy).

Being already pregnant on receiving risk information for a fatal condition meant, for one young UK-raised woman, that her choice was already made, but in future, she said, she would not take the risk:

If you have had an affected child and you know there is a risk of it happening again, the choice is between having more children, and not having any more children, because for us it is wrong to terminate a pregnancy….In my previous pregnancies, I did not know about the risk, but now I will carry that burden of worry. I just want two children now (Mrs. M, one unaffected son, one infant death).

Having information about the likely recurrence of a moderately-severe, variable and to some extent manageable condition when it was diagnosed simultaneously in a young woman’s first child and in her husband (in a late-onset form), after his arrival in the UK, did not prevent this couple from having two more children:

You can’t unmarry someone you are married to, but you can think about treatment, and you can think about the future of your children. We would want to be included in their choice of partner and we would like to arrange their marriages if we can but it would be wrong to arrange their marriages in the knowledge that you are putting the health of their children at risk (Mrs. Z., two unaffected children, one affected).

#### Postponers

A fifth of the couples were postponers, most of whom had no unaffected child. Two young UK-raised women married to cousins from Pakistan had distressing pregnancies after fatal abnormalities were diagnosed. One of these mothers had previously opted for a termination; the other had a stillborn baby with major spinal and limb abnormalities:

The doctors said I should wait at least six months before trying again, but I am not thinking of having another baby as soon as that. When I do, I will go for the amiocentesis. I said no to it last time, but I don’t want to go through this again (Mrs. Y).

Eight postponers were currently preoccupied with caring for living children affected with low to high-severity problems. A Pakistani graduate whose first child’s learning difficulties and behavioural problems caused her considerable anxiety and embarrassment said she would only have a second baby if the doctors could guarantee an unaffected child, but no test was available. One couple waited 9 years before having another child after their second child was diagnosed with a serious metabolic condition, and even then the pregnancy was unplanned and worrying, as there was no early prenatal test:

Mr. O: We were told that there is a one in four chance of having another child with this problem.

Mrs. O: Three out of four.

Mr. O: No, one out of four.

Mrs. O: Well, the point is, we did not have another child for a long time…To be honest, after he was born, I had no intention to have another baby because I had quite high chances, and that is another reason for our slow time to have another baby, 9 years and that was a long time. We just did not want to think about it. And it is not fair to the children either is it – he is like suffering at the moment. If you can avoid it, then it is better to – I mean, that’s how we look at it. If there was a test, that could tell you in the early stages… then we might have considered it, we might have gone for it, but there was not [any test], so we did not have another child for all that time.

Two couples could be offered a prenatal test, but declined it, feeling that they could not cope with another pregnancy; they were too preoccupied with the shock of their experiences of the birth, the diagnosis and of caring for their affected children.

#### Managers

Six couples were initially risk managers. Three couples had two or more unaffected children before having an affected child. The mothers in these cases had made the risk-related decisions. For example, an older, Pakistan-raised woman with two unaffected children was pregnant, and her husband was in Pakistan, when her young son’s problems were diagnosed as a high-severity syndrome marked by behavioural difficulties and physical problems. She opted for a prenatal genetic test reasoning that she could not cope with another affected child. Similarly, an older Pakistan-raised woman negotiated with her British-raised husband his agreement to mutation research (requiring blood samples from both parents and the affected child) to enable prenatal testing for the low-severity condition affecting their youngest child, and subsequently for carrier testing of the unaffected children. Her husband had been initially sceptical of their risk, thinking that with one affected child, ‘we already had our unlucky chance’, but given her husband’s health problems and the family’s fragile socio-economic position she did not want to risk another affected child.

Three couples who opted for risk management had no unaffected child, having lost a baby to a fatal condition. In these cases the UK-raised husbands negotiated prenatal mutation research and testing, their wives being relatively new to the UK with less knowledge of English and less involvement in the clinical processes. When asked if she would agree to an abortion if the test showed another baby with a fatal condition, one of these mothers responded, ‘I heard 3 in 4 chances because it is in the blood line. Only God knows everything, and he decides how it will be. If the test shows the same condition, we will do what the doctors say’.

#### The exempt

Eleven couples were categorized as exempt, that the risk was not applicable in their current circumstances. One case concerned a young couple in which only the wife was interviewed; she was currently being treated for a serious illness, was not thinking of having more children and subsequently separated from her husband, taking her affected child with her. The other couples were all older and said that they had completed their families. The conditions were in seven cases of low and in three cases of moderate severity, and in all but two families happened to affect the youngest child. Three were given a firm recessive diagnosis and the others were given a lower or uncertain recurrence risk, with implications for any children of the affected child, and the possibility, in principle of a genetic test. This was, however, a remote concern for all of these parents; as one father commented ‘we will deal with it when the time comes’. The Pakistan-raised mother of a twelve year old with a recessive condition indicated her awareness of the longer-term risk implications, by commenting ‘we won’t marry her in the family’, adding that arranging carrier tests for her unaffected children would be unnecessary because ‘marrying cousins is not part of our family tradition’.

### Change in risk responses

By the end of the fieldwork, the numbers of risk-takers and postponers had dropped (by 25% and 40% respectively) and the number of managers had more than doubled (Table 3b). Fifteen couples’ responses changed. A closer look at these cases offers some insights into the factors, processes and reasons informing these decisions. Over this time, six more fatally affected infants were born and the mutations causing two conditions were identified.

#### From postponing to risk-taking

After first delaying, three couples decided to try again without worrying about risk management. Two of these couples had previously had first pregnancies in which fatal conditions were observed on ultrasound: one had resulted in a difficult stillbirth, the other in a distressing termination that the Pakistan-raised husband had opposed, believing ‘the doctors are not always right’, but his wife’s sisters and parents had supported on the grounds that the baby would not have a life, even as a disabled child. Both couples were now decidedly ambivalent about risk management in pregnancy, although a prenatal test was available for one condition. The third couple had delayed because of their first child’s behavioural problems but unexpectedly became pregnant and had an unaffected daughter. As the mother, a graduate from Pakistan, explained:

Having this child has been such a different experience that it has changed my view of risking another pregnancy. I am no longer so afraid, and I want another child. I now think I want a big family. I had such a terrible time with my son, but realise now that it does not have to be so bad, and I really want my daughter to have a sister. I am so close to my own sister, she is really my only close friend – I have not made many friends in England and speak to my sister once a week on the phone to Pakistan. In Pakistan, all my university friends are college or university lecturers or teachers now, but I left my certificates in Pakistan and gave it all up in order to come to Britain to start a new life. Here I have nothing, only the children (Mrs. G).

#### From postponing to managing

Two young couples became risk managers after postponing. Both had only one child with high-severity problems and a low life expectancy. Knowing that their child was unlikely to live much longer and that a prenatal test was available, one of these couples opted for a prenatal test that showed an unaffected child who was born a few months before the affected child died. The other couple began to consider having another baby once they felt they were coping with their affected child, who has no intellectual delay but has had many surgical admissions:

If it is the same problem they can tell at 10–12 weeks by taking fluid from the stomach [by amniocentesis]. As you know, you were there, the doctor said there is a danger from this test that you will lose the baby, and so you have to decide before you have the test if the baby’s got the problem what would you do? It was very difficult to say, then, if I would keep it or not. Now I think that I would rather not have it. She said if I want to keep the baby there is no point of doing the test. Yes, I would want to know another time. If it does have the same problem, I would do something about it. I don’t want to go through the same problems that I have now (Mrs. R).

#### From risk-taking to managing

Four couples at risk for fatal or high-severity conditions, having previously had affected children, considered ending a subsequent pregnancy in the absence of a prenatal genetic test. One of these couples, a young Pakistan-raised mother married to a man educated in an Islamic seminary in the UK (‘he never went to an English school’, his wife said), who had two living children with a high-severity condition and two unaffected children, opted for two subsequent terminations because they felt they could not cope with another affected child. They had taken advice from a Muslim scholar who said that abortion under these circumstances was acceptable. On the other hand, another young couple sought but failed to find any religious justification for abortion on grounds of fatal abnormality, and so had experienced a traumatic birth and neonatal death; they later learnt that some Islamic schools of thought permit abortion for fatal abnormalities, and so proceeded with their next pregnancy in the light of this knowledge.

An older Pakistan-raised couple with two unaffected children who had also lost three infants to a fatal condition, having taken the risk twice, booked a termination for their sixth pregnancy, in the absence of a prenatal test, but changed their mind the day before the appointment. The wife explained what her mother-in-law had told her that she, too, had ended a pregnancy, in Pakistan, but subsequently her two unaffected children developed mental illnesses for which she blamed herself for lacking the courage to care for whatever kind of baby God gave her. ‘This gave me the strength’, the wife said, ‘to cancel the appointment’.

For the fourth couple, with one unaffected and one affected child, their experience of the condition seemed the overriding motivation for considering termination in a subsequent pregnancy. Their severely-affected still-living child had endured many surgical operations. The husband felt it would be wrong knowingly to bring another child into the world to face such suffering, and that termination ‘would be a medical decision, not a religious one’. When the next scan indicated no abnormalities, the couple declined amniocentesis, reasoning probabilistically that the risk of spontaneous abortion would now needlessly augment the risk:

The syndrome risk is 25% affected and 75% not, but the chance that this baby has [the syndrome] now looks lower than 25% because the scans show no abnormalities. I would not want to tip the balance of risk any more in the other direction or add any new worries about this pregnancy (Mr. P).

#### From risk-taking to managing, postponing and other strategies

Four couples who had experienced repeated infant deaths or stillbirths were enrolled in mutation research by the end of the fieldwork in the hope that a genetic test would become available. Doctors also discussed with them the options of adoption, which raised concerns about paternity, and gamete donation, which raised concerns about confidentiality and anonymity. Meantime, one couple took the risk again and had an unaffected child. For one of the other couples, a genetic test became available for one of the two conditions for which they were at risk. Opposed to abortion and to an invasive prenatal test with a risk of spontaneous abortion, the parents agreed to a postnatal test to inform treatment for the baby should it be affected. In their previous (third affected) pregnancy, they had welcomed being forewarned of the other genetic problem, observable at four months from ultrasound:

You mentally prepare yourself. It would be better to know. Nothing can prepare you for losing a child even after 2 and a half hours but at least it wasn’t so bad as it might have been because I already knew there was a problem (Mrs. L).

For the third couple, taking the risk again was medically contraindicated because all three babies had been delivered by Caesarian section. If a woman has had two Caesarians, doctors are unwilling to perform another if the baby will be stillborn or die neonatally. The husband took the decision; his wife accepted it sadly:

I do not want to have any more children for some time. I have gone through this 3 times - 3 Caesarians, 3 infants in intensive care, two of them dead. As a father, I do not want to have to suffer again. My main concern is what a child like that has to go through. If it was my first child, it would be different. It is having had him [one healthy child] and then having the next two and losing them – that’s what makes you think twice … I would not want to bring another child like that into the world… A 3 in 4 chance is high, but people don’t like to think about that….We have been today about birth control. After this, we do not want to have more children. Not for 5 or 6 years at any rate. My wife is very upset about this. She feels ashamed that she cannot produce healthy children. She feels she is to blame, that she is no use – she cannot perform the function for which she is here. I do not blame her. It is a cultural thing that she feels like this (Mr. J).

Having lost three infants to a fatal condition, the husband in the fourth couple thought something non-genetic must be involved, because their bad luck should have ‘run out’:

After the first, it was unlikely to be this again. Then after the second, we were told there is a risk – but it is okay to try again. Then it happened again. It has come again and again. In the probability sense, the chance should have been less and less, we thought is would be less and less [each time]. They ruled it out, after each birth; each time, we were told it is just bad luck. But the next child was affected, and the next. I think there must be some other underlying factor (Mr. N).

His decision was to delay:

We have decided we won’t have any more kids until we have an answer to my question about prenatal diagnosis. We don’t want to go through this again. We feel it is a big risk, to bring a child into this world to experience such pain. And there is the 9 months of pregnancy to consider, as well as what happens after the birth. We waited 2 or 3 years after [our first child] was born, thinking we should space the children. If we have known then, we would have stopped … for 3 or 4 years and not had more (Mr. N).

Yet, finding anonymous gamete donation unacceptable, he also enquired about egg donation from his wife’s sister but was advised that she could be a carrier for the condition and that egg donation is technically more difficult, risky and less successful than sperm donation, which he considered unacceptable. At this point, he also considered a strategy for taking the risk many times simultaneously through fertility treatment for his wife, if unavailable through the National Health Service (this couple has no fertility problem) then privately or abroad. He reasoned probabilistically that, given a 1 in 4 risk with each conception, simultaneous multiple conceptions will make a good outcome more likely, like buying many lottery tickets:

I am 29 now and my wife is nearly 32. Time is passing and we want a family – we only have one child, and have lost three. That is why I want to go for fertility treatment – that way we would have our family all in one go and maybe one child would be affected and would eventually die but two wouldn’t – then we could be happy, we will have a family (Mr. N.)

#### From managing to postponing or exempt

Two initial managers changed their strategies. One young childless couple, at risk for a fatal condition, delayed another pregnancy after their second pregnancy miscarried following a genetic prenatal test, which they had agreed to with a view to termination ‘for medical reasons’ if the pregnancy was affected. The UK-raised husband, who had negotiated with the doctors because his wife speaks little English, now understood his risk as ‘high’, as a ‘3 in 4’ chance; his wife, wanting a baby, was less sure about postponing but decidedly ambivalent about prenatal risk management, including even the use of ultrasound scans, saying that ‘only God knows if a baby will be healthy or not’.

The second case concerned the older woman whose prenatal test in her fourth pregnancy had shown an unaffected baby. She continued to worry about the risk of having another child with a high-severity condition so opted for sterilization after the birth; ‘I did not want to take the risk again’. Her decision can be seen as a move to become exempt from risk; with four children, three unaffected, she had, by now, effectively completed her family.

## Discussion

This study describes British Pakistani adults’ responses to medical information about genetic risk in terms of risk-acceptability (risk-takers) and non-acceptability (postponers, managers and the exempt). The analysis shows a range of responses to statistical risk information that are shaped by social circumstances and moral considerations; it illustrates a range of strategies of ‘responsible parenting’ (Kelly, 2009). The risk-takers, for example, tended to downplay numerical risk, referring to the fact that they already had a healthy child or children, the rarity of the condition and the stigmatizing public health discourse on cousin marriages (Shaw, 2009). The gambling fallacy, that after many losses you are due a win, or vice-versa, has been identified as a common health professionals’ bias in the interpretation of statistics whereby connections are made between statistically independent events (Gigerenzer, 2002, 2008). In the present context this may reflect overarching desires for a child, more children or to give a single child a sibling. Likewise, remarks by some older adults and younger Pakistan-raised women that reproductive outcomes are ‘up to God’ may also be interpreted as idioms for expressing risk-acceptability rather than indications of necessarily fatalistic or religious beliefs.

Medical anthropologists have examined risk perception in social and cultural context, identifying agency in decision-making and demonstrating the logic of risk-taking in relation to other risks that must simultaneously be negotiated (Caplan 2000; Jenkins et al., 2005; Sobo, 1995). In arranged consanguineous marriages, for example, the socio-economic and emotional risks associated with marrying outside the family may, consciously or not, be balanced against the elevated risk of recessively-inherited genetic problems in children (Shaw, 2009). This study demonstrates the limitations of focussing only on numerical risk: in prenatal decisions the probability of a genetic problem in a pregnancy may, consciously or not, be balanced against the social and emotional risks of childlessness, of spontaneous miscarriage following an invasive test, or of abortion where this is regarded with moral ambivalence or censure by kin and within the local moral and religious community. Thus, the response of the woman who had lost three babies to a fatal condition but declined an abortion at the eleventh-hour could be described as risk-averse; for her, a termination may have been socially and morally riskier than having another affected child.

This study also shows that risk responses change over time with subsequent reproductive experience, with the biggest change being towards risk management, capturing a dynamic missing from snapshot studies. It also reinforces the observation, made in a comparison of attitudes to prenatal risk management of American parents of children with cystic fibrosis with their reproductive behaviour five years later, that change can occur in either direction: towards or away from risk management (Sawyer et al., 2006). In this study, some of those categorized as currently exempt were nonetheless aware of the long-term implications for their children, and one risk manager had arranged carrier tests for her unaffected children. This has relevance for understanding processes of risk-communication and disclosure with the wider family (Shaw & Hurst, 2009). Those categorized here as risk-takers may, in the long, term become more risk-averse in relation to the marriages of their own children. Future studies might therefore explore short-term versus longer-term responses.

This study adds to evidence from national and international studies that couples’ responses to invitations to risk management are mediated by their previous reproductive history and their perceptions of the severity of the disease rather than by religion or culture (Ahmed, Atkin et al., 2006; Ahmed, Green et al., 2006; Ahmed et al., 2008; Hewison et al., 2007; Zlotogora, 2002). Migration history, usually taken as a proxy for acculturation towards ‘western’ norms, here is not a good predictor of attitudes; some young Pakistan-educated adults in this study had greater awareness of the range of Islamic opinion regarding prenatal diagnosis and abortion, including the view that permits abortion under certain conditions, than their UK-raised counterparts (Atighetchi, 2007, pp. 91–133; 250–254; Shaw, in press). The study also shows a high degree of reflexivity on the part of young adults in relation to their personal experience of risk, their cultural and religious expectations and the information received from doctors, demonstrating their ability to negotiate social change (Archer, 2003, 2007).

Women rather than men are often the subject of studies of prenatal testing, or contraceptive use (Ahmed, Atkin et al., 2006; Ahmed, Green et al., 2006; Ahmed et al., 2008; Dormandy et al., 2005; Sobo, 1995). Men as well as women were interviewed in this study. Some of the young UK-raised husbands with Pakistan-raised wives with poor English were very directly involved in negotiations with their doctors and their wives. Expressing concerns about gamete donation shared by some other South Asian couples (Culley & Hudson, 2007), one husband considered taking his quest for an unaffected child overseas to where less restricted infertility treatments may be available. On the other hand, there was also a tendency to see genetic risk as ‘women’s business’. Some older Pakistan-raised women with unaffected children were the main decision-makers regarding genetic risk, perhaps reflecting the authority a woman traditionally acquires once she has secured her status as a mother. The findings here thus support recent research showing how genetic responsibility both challenges and reinforces the traditional gender division of labour (Reed, 2009).

In this study, the categorization of risk responses posed some problems. The focus was on action rather than attitudes, but two couples were categorised as risk managers on the basis of the interviews without having another pregnancy during the fieldwork. Attitudes and behaviour are analytically distinguishable, and research shows that women of ethnic minority and low income groups less likely to act in line with their attitudes, for a range of different reasons (Dormandy et al., 2005). During the fieldwork, the researcher spent time discussing these issues in Urdu with women whose English was poor, but without continuing support these women may be less likely to act in line with attitudes. This study is confined to one ethnic group in a district which has the second largest Muslim population in southern England outside London. However, the fact that the majority of the Pakistani-origin families referred to the genetics clinic over the study period were recruited into this study provides high ascertainment of the range of genetic conditions in this population and of couples’ responses to risk information.

Theorists of risk as a cultural preference have portrayed risk-taking as the transgression of dominant cultural norms (Douglas, 1992; Douglas & Wildavsky, 1982). From this perspective, Pakistani couples’ risk-taking implies the maintenance of minority ethnic-cultural boundaries with respect to the majority society’s marital and reproductive norms, a view perpetuated in media discourse on genetic risk in cousin marriage. This study challenges such a view by demonstrating, first, the heterogeneity and dynamism of action within a single ethnic-cultural group, second, the engagement of its members with a range of modern forms of risk negotiation and, third, the many similarities between the findings of this study and those from studies conducted in other populations.

## Figures and Tables

**Table 1:** Socio-demographic characteristics.

Parents’ education by gender and age at date of first interview							
Age	P1	P2	P3	UK1	UK2	UK3	Totals
Men							
20–24	0	2	0	0	0	1	3
25–34	0	10	1	3	12	7	33
35–44	0	0	0	3	1	1	5
45–54	2	3	0	1	1	0	7
55–64	1	1	0	1	0	0	3
Totals	3	16	1	8	14	9	51
Women							
20–24	2	4	3	0	4	0	13
25–34	6	9	4	0	6	0	25
35–44	5	2	0	0	0	1	8
45–54	2	2	0	0	0	0	4
55–64	0	1	0	0	0	0	1
Totals	15	18	7	0	10	1	51

PI: mimimal/primary education in Pakistan
P2: secondary education in Pakistan
P3: college/university education in Pakistan
UK1: 3–6 years of education in UK
UK2: compulsory secondary education (to age 16) in UK
UK3 college/university education in UK.

	No.
Family origins in Pakistan	
Azad Kashmir	23
Punjab	24
Karachi	3
Not recorded	1
Total cases	51
Parental consanguinity	
Consanguineous	
First cousins	34
Double first cousins	2
More distant	11
Total	47
Unrelated	4
Total couples	51
More children wanted	
Yes	25
Maybe	3
No	21
Total couples	51
Other affected family members	
Yes	8
No	43
Total couples	51

	No. couples
No. of affected children/pregnancies	
0	3
1	39
2	6
3	3
4	0
5	0
6	0
	51
No. of unaffected children	
0	15
1	10
2	13
3	6
4	2
5	1
6	2
	51

**Table 2:** Genetic diagnosis and risk information.

Severity of conditions observed	No. conditions
Fatal	16
High	14
Moderate	6
Low	18
Total observed conditions	54

**Fatal**: includes known and unknown lethal conditions associated with inter-uterine death,stillbirth, neonatal death, and death in first few years of life, such as hydrocephaly, infant polycystic kidney disease.
**High**: includes known and unknown dysmorphic syndromes associated with a range of physical and usually also intellectual problems and low life expectancy, such as Fanconi’s anaemia, San Fillippo syndrome, Robert’s syndrome, Otahara syndrome, I-cell disease, Sotos syndrome, Prader–Willi syndrome, Laurence-Moon Bardet-Biedl syndrome.
**Moderate**: includes severe but late-onset conditions such as forms of ataxia; serious learning difficulties without physical problems, and syndromes associated with physical and intellectual problems of variable severities such as Neurofibromatosis type 1.
**Low**: includes learning difficulties with no or minor physical problems and physical problems without learning difficulties and amenable to some degree of treatment, such as Albinism, Deafness, Congenital Adrenal Hyperplasia, cleft palate, imperforate anus, genital ambiguity and forms of chromosomal mosaicism including McCune Albright syndrome.

Risk information	Mode of Inheritance	
1 in 4 (definite/most likely/likely)	Autosomal Recessive	33
7 in 16	Autosomal Recessive	3
1 in 2	Autosomal Dominant	1
Unlikely, but risk to children of affected child	Dominant sporadic	4
1 in 2	Chromosomal1	1
Unlikely, but risk to children of affected child	Chromosomal sporadic	4
1 in 4 (highest likely)	Unknown	5
	Total cases	51

**Table 3:** (a) Initial risk responses. (b) Initial and subsequent risk responses.

(a)	Severity				Test available		Unaffected children		
	L	M	H	F^a^	Yes	No	Yes	No	Total
Takers	6	2	8	8	3	21	19	5	24
Postponers	3	0	5	2	2	8	2	8	10
Managers	1	0	2	3	3	3	3	3	6
The exempt	8	3	0	0	2	9	11	0	11
Total	18	5	15	13	10	41	36	15	51

(b)	Takers	Postponers	Managers	Exempt
Initial	24	10	6	11
Later	19	6	14	12

a 3 families with 2 fatal conditions.
